# Priority setting in primary health care - dilemmas and opportunities: a focus group study

**DOI:** 10.1186/1471-2296-11-71

**Published:** 2010-09-23

**Authors:** Eva Arvidsson, Malin André, Lars Borgquist, Per Carlsson

**Affiliations:** 1 Department of Medicine and Health Sciences, Centre for Medical Technology Assessment, Linköping University, Linköping, Sweden; 2 Department of Primary Health Care, Kalmar, Sweden; 3 Centre for Clinical Research, Falun, Sweden; 4 Department of Medicine and Health Sciences, Division of General Practice, Linköping University, Linköping, Sweden; 5 Department of Medicine and Health Sciences, Centre for Priority Setting in Health Care

## Abstract

**Background:**

Swedish health care authorities use three key criteria to produce national guidelines for local priority setting: severity of the health condition, expected patient benefit, and cost-effectiveness of medical intervention. Priority setting in primary health care (PHC) has significant implications for health costs and outcomes in the health care system. Nevertheless, these guidelines have been implemented to a very limited degree in PHC. The objective of the study was to qualitatively assess how general practitioners (GPs) and nurses perceive the application of the three key priority-setting criteria.

**Methods:**

Focus groups were held with GPs and nurses at primary health care centres, where the staff had a short period of experience in using the criteria for prioritising in their daily work.

**Results:**

The staff found the three key priority-setting criteria (severity, patient benefit, and cost-effectiveness) to be valuable for priority setting in PHC. However, when the criteria were applied in PHC, three additional dimensions were identified: 1) viewpoint (medical or patient's), 2) timeframe (now or later), and 3) evidence level (group or individual).

**Conclusions:**

The three key priority-setting criteria were useful. Considering the three additional dimensions might enhance implementation of national guidelines in PHC and is probably a prerequisite for the criteria to be useful in priority setting for individual patients.

## Background

Priority setting can be defined as making a choice based on a ranking process, although occasionally the term is used as a synonym for rationing or resource allocation [1,2]. Prioritising takes place in all parts of the health care system where demands and needs exceed resources. Decisions on priority setting are made at different levels [3,4]. General policy decisions are made at national and regional levels, as are comprehensive decisions on resource allocation, systems for financing providers, and national guidelines including priority setting for management of common diseases. This paper addresses primary care, where priorities are set on a practical, individual level.

In Sweden nearly all health care is publicly financed through taxes, and the entire population is insured. Each primary health care centre is funded based on the number of patients linked to the centre, and in some instances the number of visits determines a small portion of the budget. Hence, each primary health care centre receives a limited, fixed budget to serve its patients. GPs work in close collaboration with district nurses. Most appointments with a GP are preceded by a telephone call to a nurse that decides whether to schedule the patient to see a GP, or whether advice by telephone will suffice. Prioritising takes place at an individual level where staff decide who will be given an appointment and who will have to wait. Decisions concerning the choice of interventions and treatments for individual patients are made during the consultations [5].

Priority setting in primary health care (PHC) is important because its outcome has significant implications for health care costs and outcomes in the system as a whole [6]. Several countries with publicly financed health care systems have formulated ethical principles and criteria for priority setting in health care [1,5,7,8]. In Sweden, guidelines for priority setting are based on three key criteria: 1) severity of the health condition, 2) patient benefit, and 3) cost-effectiveness of the medical intervention [9]. National guidelines for managing common diseases are developed mainly by medical experts from hospital settings and address single diseases or disease categories [10,11]. The guidelines have been implemented for policy decisions and priority setting for individual patients in secondary care [12].

Methods for priority setting in primary health care have been suggested [4,13,14], but there are few practical examples of systematic priority setting in PHC [15]. One explanation could be that the priority setting tools were constructed in hospital settings with limited initial involvement from GPs and are therefore difficult to use in PHC. No studies were found that analysed how GPs or nurses in PHC used or perceived the key criteria.

## Objective

To analyse how GPs and nurses perceive the application, in their clinical practise, of the three key priority-setting criteria: 1) severity of the health condition, 2) patient benefit, and 3) cost-effectiveness of the medical intervention.

## Methods

Focus groups were chosen for data collection because this method is effective in exploring how people think and reason in certain situations and why they think the way they do [16]. The interaction between participants enforces exploration and clarification of their views [17].

To assemble groups experienced in using the priority-setting criteria, participants were recruited from GPs and nurses working in four purposely selected primary health care (PHC) centres in rural and urban municipalities in southern Sweden where a study on priority setting had been conducted earlier. In the earlier study, which included 4300 patient contacts during 2 weeks, the GPs and nurses answered a questionnaire where they used the three key priority-setting criteria and estimated the severity of the health condition, the expected patient benefit, and the cost-effectiveness of the planned intervention. Concurrently, they were given written information about the three key criteria (Table 1). The focus groups met 5 months after the staff had participated in the earlier study.

**Table 1:** Schematic description of the key criteria to be considered in priority setting.

Severity of the health condition	Patient benefit	Cost-effectiveness of the intervention
**Current health condition**	**Effects on current health condition**	**Direct costs**
- suffering	- suffering	- health service interventions,
- functional impairment	- functional impairment	- other measures, e.g. travel
- quality of life	- quality of life	
		
**Risk of**	**Effects on risk**	**Indirect costs**
- premature death	- premature death	
- disability/continued suffering	- disability/continued suffering	
- lower quality of life	- lower quality of life	
		
	**Risk of side effects and severe complications from intervention** ... in relation to the benefit of the intervention	

All staff members who participated in the earlier study (in total 24 GPs and 54 nurses from the four PHC centres) were invited to participate in the focus groups. Written information was sent to each of them. The focus group sessions were held at the primary health care centres, and all GPs and nurses on duty the day of the focus groups (16 GPs and 15 nurses) took part. The GPs included ten men and six women. Four of them were under 50 years of age, and all but two had more than 10 years of experience. All nurses were women, six were under 50 years of age, and three had less than 10 years of experience. Eight focus groups were held (one GP group and one nurse group in each of the four PHC centres). Each group consisted of two to six participants. Two of the authors, EA and MA, jointly conducted the discussions in the focus groups. The sessions lasted approximately 1.0 to 1.5 hours. The topics discussed concerned the participants' perceptions of:

• the severity of the patient's health condition

• the effect or patient benefit of the (planned) intervention

• the cost-effectiveness of the (planned) intervention.

The focus group discussions were audio-taped verbatim and transcribed unedited for subsequent analysis.

Initially the transcripts from the focus groups were read several times by EA and MA to obtain an overview of the material. Subsequently meaning units in the text were identified and manually sorted into groups according to the topics from the main questions in the focus groups, i.e. the three key priority-setting criteria. Concurrently, themes that recurred in all of these groups were identified and coded. In the next step, categories were formed by analysing and combining coded units [18-20]. The categories were discussed in the research group during several meetings. Disagreements were resolved through consensus.

The Research Ethics Committee of Linköping University approved this study. It was supported by FORSS (Council for Research in Southeast Sweden, the county councils of Jönköping, Kalmar, and Östergötland, and the Faculty of Health Sciences, Linköping University 2005).

## Results

The GPs and nurses found the key priority-setting criteria to be useful, reporting that these criteria made them think in a new way when prioritising. One GP said:

"What's new, it's these two - patient benefit and cost-effectiveness. We always thought about severity. But we didn't consider so much whether the patient would actually benefit from coming here."*(GP 3, Group 3)*

They described how using the key priority-setting criteria stimulated them to consider carefully the value that an intervention actually had for the patient:

"I think this is more appropriate. Without this, my impression of a consultation plays a major role: 'How was the contact? What actions did I take?' This way is more systematic: 'The disease is diabetes. It's a serious disease that needs to be managed properly even if really I don't do much more than find that it looks pretty decent and renew prescriptions.' When I adjust my subjective impression of not doing so much, so much effort, it is adjusted in a more systematic way." *(GP 3, Group 4)*

The staff were more detailed in discussions concerning the severity of the health condition and patient benefit than they were about cost-effectiveness. Most reported difficulty in applying the cost-effectiveness concept. Some staff members did not want to think about the costs of health care at all.

"It's terrible to say, but this thinking about economy ... even if I know ... What we learned was nursing, to take care of patients and diseases. And I am supposed to think about cost-effectiveness and health benefits and .... No, this is difficult." *(Nurse 2, Group 8)*

In spite of the difficulties in applying the concept of cost-effectiveness, it was considered to be an important criterion in priority setting.

"This is what it's all about, how we should use resources in the best way". *(Nurse 1, Group 6)*

Three different categories that describe three additional dimensions in priority setting were identified: 1) viewpoint (medical or patient's), 2) timeframe (now or later), and 3) evidence level (group or individual).

### Viewpoint - medical or patient's

Throughout the focus group discussions two aspects facing the GPs and nurses were apparent: 1) the need to treat the illness and 2) the need to understand the individual patient. These two aspects were often described as the medical viewpoint and the patient's viewpoint.

"I've thought about this, and there are two ways of looking at it. On one hand, there is the medical severity, the seriousness of the disease. But then, there is also the severity of the trouble the disease causes the patient. Although I know that it's harmless, it may be very troublesome to the patient just now. So I have to try to consider both aspects." *(GP 3, Group 4)*

The GPs referred to these two viewpoints with regard to both severity of the condition and benefit to the patient. The medical viewpoint was based on the use of medical knowledge to estimate the seriousness of the condition and the benefits and risks of different interventions.

"I tried to think of the disease itself, as a disease, and not so much really about how the patient experienced it." *(GP 2, Group 3)*

(See also Table 2, Nos. 1-3)

**Table 2:** Statements from Swedish focus groups with general practitioners (GP) and nurses (N) concerning key priority-setting criteria (severity of the health condition, patient benefit, and cost-effectiveness) that exemplify the category of *viewpoint *(medical or patient's).

Viewpoint	Severity of the health condition	Patient benefit	Cost-effectiveness of the intervention
**Medical viewpoint**	1. A melanoma, for example, a small melanoma which the doctor sees as very serious, may be seen by the patient as nothing more than a normal birthmark. *(GP 6, Group 4)*	3. Yes, it was a case of depression. I felt that something concrete occurred there. *(GP 3, Group 3)*	4. Elderly patients who come to collect their medicines, talk a little, and get support. Seeing me here makes them feel secure. And when I do this it saves on other care facilities. It saves doctors' appointments, maybe visits to the hospital, and lots of other things and the patients still feel good. *(N 1, Group 8)*
	2. I had some inadequately controlled diabetes patients who did not see this as a major problem. *(GP 2, Group 3)*		

**Patient's viewpoint**	5. It may be trifling matters that reduce the patient's quality of life, which, as a member of the medical staff you may not consider to be so serious. However, it may be serious for the patient. They experience it as more of a problem than we do. *(N 2, Group 5)*	6. When a patient suffers from globus sensation they assume that they have cancer of the throat. You can explain various mechanisms to the patient and different methods of treatment, but not perform any intervention. But the patient is often very relieved when they leave the surgery. *(GP 3, Group 1)*	8. Medicated stockings that are used week after week. The patient insists on having them even if... well there are some small changes in their skin condition. It cannot be very cost-effective to go home to them to put them on. Admittedly the patient buys them him/herself, but that is just money. They insist on having them, but it's not certain that they are of any help. *(N 3, Group 5)*
		7. It is also of great importance. I mean if they feel very satisfied it has been a great benefit. *(N 2, Group 6)*	

The statements are numbered in the order of their reference in the text.

The patients' viewpoint was based on the GPs' or nurses' estimation of how patients experienced their symptoms, how worried the patients felt, and how satisfied the patients would be with the interventions.

"You may be convinced that it's a very mild condition ... but it may still reduce the patient's functional ability due to anxiety. *"(GP 1, Group 3)*

(See also Table 2, Nos. 5-7)

Some GPs considered the medical viewpoint more important, or even the only viewpoint that should be considered, especially when estimating patient benefit. However, most of the GPs found it important to take the patient's viewpoint into account and balance the two viewpoints in estimating the severity of the condition and expected patient benefit. Most nurses considered patient satisfaction to be an important aspect of patient benefit, but many GPs said that patient satisfaction is not directly related to the actual benefit of the treatment given.

"The fact that a patient is satisfied only means that you have met his/her demands, but that does not necessarily mean that they have received any special benefit from the treatment." *(GP 1, Group 2)*

(See also Table 2, No. 7)

General practitioners and nurses also used the two viewpoints when estimating cost-effectiveness, albeit more indirectly (Table 2, Nos. 4, 8).

### Timeframe - now or later

In most cases the GPs and nurses estimated the severity of the patient's condition from the patient's well-being at the time of the consultation and not by considering future risks:

"I would consider the case of a diabetic under strict surveillance to be of only moderate severity. Perhaps I would not need to perform any intervention during the check-ups since it was already under control. But in the case of a patient who is going downhill, if I could intervene to stop that process in any way it would, of course, be a condition of high severity." *(GP 3, Group 1)*

General practitioners and nurses found it relatively easy to estimate the severity of a condition in patients with obvious symptoms of a well-defined, usually acute, disease. These types of conditions were often considered more severe than asymptomatic chronic conditions. At times, the severity of a condition was equated with how soon the patient needed an appointment, i.e. acute diseases were considered more severe than chronic diseases that needed check-ups but could wait another day.

"A patient that comes in with severe sepsis is in a critical state and will die if he or she does not receive treatment within a matter of hours. Of course this patient must be given highest priority." (*GP 3, Group 4)*

(See also Table 3, Nos. 1, 6).

**Table 3:** Statements from Swedish focus groups with general practitioners (GP) and nurses (N) concerning the key priority-setting criteria (severity of health condition, patient benefit, and cost-effectiveness) that exemplify the category of *timeframe*.

Timeframe	Severity of the health condition	Patient benefit	Cost-effectiveness of the intervention
**Now**	1. It is simple if a patient comes in with an acute heart attack. Or if it's a case of ileus or some other acute medical condition. But if it's a chronic condition it is harder. *(GP 1, Group 1)*	2. You get certain patients who you can deal with before they leave the room. And it doesn't take long either. There aren't so many of them, but when it is impacted wax, or something like that. *(GP 5, Group 4)*	4. I was just thinking that I wish that one of my patients would suddenly stand up and say: "Now" he would say, "I have taken my last drag. Now I have quit. Now I have quit smoking, right now." *(GP1, Group 1)*
			5. A simple wound that requires stitches not to leave an unsightly scar... and may be dealt with in a few minutes. *(GP1, Group 1)*
		3. There were patients I checked to see if they had a wound infection. Then, it was really important to see them so they could perhaps have penicillin or something. *(N 2, Group 6)*	

**Later**	6. A diabetic who may perhaps suffer from gangrene in five years' time. Well, it would be difficult to call that a highly prioritised patient today. *(Gp3, Group 4)*	7. In the case of chronic illnesses, the patient is often aware of the situation... well, one has talked to them before and explained that there's not a lot more that can be done. *(GP 2, Group 1)*	8. It is really difficult. When do we consider that hypertension treatment is cost-effective? If we treat a woman of around 40 for mild hypertension it isn't really cost-effective. *(GP 2, Group 1)*
			9. If there's something that the patient comes to see us for, that at a later stage... or in other words, if you delay, it may be much more expensive. *(N 1, Group 7)*

The statements are numbered in the order of their reference in the text.

Likewise, estimating patient benefit and cost-effectiveness of an intervention was found to be easier when the intervention was uncomplicated and yielded a quick result that could be easily perceived or measured:

"If someone comes in with a sort of asthma characterised by severe rhonchi and needs inhalation treatment, which will clear it up straight away." *(GP 1, Group 3)*

(See also Table 3, Nos. 2-5).

It was more difficult to evaluate patient benefit in asymptomatic patients with chronic conditions and who are at risk for future complications, e.g. diabetes or hypertension. General practitioners expressed difficulties in knowing what benefit a patient would realise in the future from a particular intervention given today.

Moreover, some GPs questioned the value of treating certain chronic conditions, e.g. hypertension. They considered treatment to be overrated, which made the estimation of patient benefit even more difficult.

"A urinary tract infection, and so on, these patients were easier for me to assess. But, for example, a yearly check-up for hypertension where blood pressure was a little too high is difficult for me because there is a greater risk of heart attack and so on, but of course not very high..." *(GP 4, Group 1)*

(See also Table 3, Nos. 7-9).

### Evidence level - individual or group

In patients presenting with common symptoms, the staff found it easy to estimate severity without questioning the resulting patient benefit and cost-effectiveness.

"Of course, if there is a diabetic with an infection I assess it a little differently than if it is a healthy person with the infection." *(GP3, Group 4)*

"I had a COPD patient who did not want to quit smoking, who I prescribed medicine to, but I wrote that it would offer a low degree of patient benefit." *(GP 3, Group 3)*

(See also Table 4, Nos. 1-4).

**Table 4:** Statements from Swedish focus groups with general practitioners (GP) and nurses (N) concerning the key priority-setting criteria (severity of health condition, patient benefit, and cost-effectiveness) that exemplify the category of *evidence level*.

Evidence level	Severity of the health condition	Patient benefit	Cost-effectiveness of the intervention
**Individual**	1. When you have a patient in front of you... well, for that patient it might reduce functional capacity even if the disease normally doesn't. *(GP 1, Group 3)*	2. If you make a home visit to an elderly patient that has had a fall, if the patient has a fracture of course there is a high degree of patient benefit, but if the patient doesn't have a fracture... the benefit isn't so great. It would heal anyway. *(N 2, Group 5)*	4. It involves a cost when patients call. I mean, it takes time and so on... But you can fix it at home yourself, your immune system will fix it! If you come here it is a cost both for you and for us. *(N1, Group 6)*
		3. For example a patient with a cold that comes to see a doctor. It isn't very important for this group of patients to see a doctor. There is little patient benefit in this case. But it may be of great benefit to the particular patient if he or she learns that he or she doesn't need to see a doctor the next time he or she gets a cold. *(N 4, Group 6)*	

**Group**	5. We have patients with chronic illnesses that are potentially, perhaps not fatal, but at least threaten the patient's quality of life and functional ability. There's a degree of this threat in most chronic illnesses. But if they are well monitored, and the symptoms are under control, I don't think I would call it extremely severe. *(GP 4, Group 1)*	6. But if you prescribe medicine for blood pressure, then you know that, at best, 15% of those you give medicine will, if they follow your instructions, be helped by it. *(GP 3, Group 3)*	7. Vaccination of the elderly against influenza, I have called that highly cost-effective, based on the recommendations of the Swedish Board of Health and Welfare. *(N 1, Group 6)*

The statements are numbered in the order of their reference in the text.

However, to estimate severity, patient benefit, and cost-effectiveness for a non-symptomatic patient with a chronic disease, the GPs had to base their estimation of risk for complications and the likely benefit of interventions on documented, population-based studies.

"Some patients benefit from it. But you can never know whether a particular patient will benefit." *(GP 5, Group 4)*

(See also Table 4, Nos. 5-7)

Applying evidence-based knowledge about study populations to individual patients took place later in the timeframe category, i.e. difficulties associated with estimating how an intervention will affect future risks and benefits.

Moreover, the GPs considered that an individual patient's compliance with lifestyle recommendations would also affect the health outcome. Hence, benefit and cost-effectiveness were dependent not only on the evidence-based intervention, but also on the behaviour of the individual patient.

"I argued that in the case of chronic patients with diabetes and such, if you can manage to convince them when they come to the surgery that there are things they should do to have a good life, then it is cost-effective time. To get them to change their behaviour patterns, getting overweight patients to understand that they must lose weight and such. Then I think that you are highly cost-effective." *(GP 1, Group 2)*

(See also Table 4, Nos. 3, 6).

## Discussion

When the nurses and GPs applied the three key priority-setting criteria to primary health care (PHC) problems, they perceived them to be relevant, but not sufficient. They described difficulties in using the criteria, and identified three additional dimensions to consider in priority setting in PHC, namely *viewpoint *(medical or patient's), *timeframe *(now or later) and *evidence level *(group or individual). Viewpoint concerns the importance of taking both the patient's and the medical viewpoint into account when assessing patient benefit and the severity of the condition. Timeframe concerns estimates based on present symptoms and benefits versus the risk of complications in the future and possible future gain from different interventions. It also indicates that patients with acute conditions and present symptoms are more likely to be given higher priority than patients with a risk of future disease progression or future complications. Evidence level concerns the individual versus the group. In the context of clinical priority setting, it indicates the difficulties associated with taking scientific knowledge acquired from studies of a group of patients (e.g. with a certain diagnosis) and applying it to an individual patient.

Our field of interest is new, and our research is explorative. However, some methodological limitations are recognised. As knowledge about the concepts of priority setting is relatively limited in PHC [15], the authors selected the focus group participants for their experience in priority setting. The fact that the focus groups were limited to the primary health care centres previously involved in a study on priority setting might have influenced the result. However, participant familiarity with the key principles for priority setting was a prerequisite for the study.

In focus groups the participants influence each other, and the data collected reflect both individual and collective norms and beliefs. In most sessions the initial statements about the key criteria reflected the instructions for their use given during the prior study (Table 1). Hence, it took some time before new dimensions of using the key criteria in PHC became apparent. The number of participants in each session created a good climate for the moderators to facilitate discussions and to give all participants the opportunity to express themselves fully. One group of nurses had only two participants, which may have reduced the effects of group dynamics.

All staff members who took part in the earlier study were invited to participate in the focus groups. Those present at work on the days the focus group sessions were conducted took part, i.e. two thirds of the GPs and one fourth of the nurses. Statements supporting all of the categories were given in nearly all focus groups, but it is possible that additional sessions with the remaining staff, certainly the nurses, would have generated further information.

The GPs and nurses worked together, and had they participated in the same focus group the differences in their perceptions might have diminished [16]. This was the reason for separate focus groups. Overall, the nurses emphasised the patients' viewpoint, whereas the GPs were more concerned with how the timeframe and the evidence level influenced the perception of severity of the condition and the effectiveness of the intervention. The GPs also gave more examples of dilemmas in using the key priority-setting criteria.

As organisational characteristics and professional roles in PHC differ between countries, some of the findings might be context-bound to Sweden, which is a limitation of the study.

Practical applicability of the criteria is important because without tools for prioritising at the individual level, there is a risk that decisions on the individual level would be made on grounds other than the nationally accepted ethical principles and key criteria. The GPs and nurses were relatively comfortable with using two of the criteria for priority setting: severity and patient benefit, both of which are familiar concepts in daily PHC work. The third criterion, i.e. cost-effectiveness, was considered difficult to use. Several reasons why GPs find it difficult to assess cost-effectiveness have been suggested, including mistrust of health economic concepts among GPs, difficulties in estimating needs as a basis for cost-effectiveness, and the conflict between health economy and the GPs' patient-centred work [21-23]. Although the GPs and nurses found cost-effectiveness difficult to estimate, several of them said it is an important criterion in priority setting. One reason for this could be a relatively high degree of cost awareness among Swedish GPs compared to other specialists, particularly in county councils with decentralised drug budgets [24].

Although some GPs did not consider the patient's viewpoint relevant when evaluating the severity of the condition and patient benefit, most of them included patient worries and expected outcome. Several studies show that physicians use factors other than biomedical criteria about patients in priority setting [25,26]. In general practice the two viewpoints, medical and the patient's, have long been emphasised [27,28]. One of the key features of general practice is its role as first-line health care. Patients wish to consult medical staff because of their anxiety about symptoms more so than about the effects of a well-defined disease [29]. A study from the Netherlands showed that in 12% of the consultations neither a specific diagnosis could be made nor were the problems explained by the patient's somatic or psychosocial context [30]. This reinforces the patient's role as an important source of knowledge [27] and is one reason why patient-centred work is considered a core competence in PHC [29,31].

The nurses emphasised patient satisfaction as a dimension in the patient's viewpoint. In contrast, the GPs argued that patient satisfaction is not always linked to what is medically appropriate and should therefore not be considered when estimating patient benefit. These opposing views of patient satisfaction illustrate conflicting interpretations of the principles used in prioritising. Health care today places greater emphasis on patient satisfaction, good accessibility, and short waiting times. Regulations on health care are undergoing change, shifting from a previous focus on the needs of patients to health care based on the legal claims underlying patients' rights [23,32]. In Sweden, the evidence-based national guidelines introduced by the National Board of Health and Welfare can be viewed as a means to deliver health care based on needs. Open discussions, e.g. on how to evaluate anxiety in patients with a harmless disease or patient satisfaction in relation to scientific knowledge, are important in this context [33].

Several authors have raised the ethical issue of how to evaluate benefit for the individual compared to benefit for a group of patients or society at large [33,34]. The conflict between the individual and group levels expressed in our study is not only an ethical conflict, but involves applying knowledge about a group to estimate the benefit to the individual. This issue could be interpreted both as a source of difficulty in practising evidence-based medicine (EBM) and as a sense of doubt about its value in PHC.

The EBM paradigm, together with ethical principles, is supposed to form the basis for priority setting. It has been described as the integration of individual clinical expertise with the best available, external, clinical evidence [35]. The application of EBM is found to be more controversial in general practice than in other specialities [36]. It has met resistance from some GPs on the grounds that practising EBM interferes with patient-centred consultation. Hence, EBM and the patient-centred method have become polarised instead of integrated [37], aggravated by the ongoing debate questioning how much of the agenda should be based on future risks instead of the patient's present concerns [38].

When discussing the risks of complications and the effectiveness of treating patients with hypertension or diabetes, the GPs expressed frustration and doubt concerning how to manage these patients. They found the evidence insufficient and the conclusions uncertain as regards the effectiveness of many common treatments provided in PHC.

The doubt expressed about the patient benefit of preventive care for chronic conditions might lead to underestimating the needs and effects of interventions for these patients. In contrast to cases involving chronic disorders, evidence based medicine was not discussed or used in managing acute conditions. Instead, effectiveness and patient benefit were often unquestioned, which could result in overestimating the benefits in these cases.

The GPs and nurses judged patient benefit based on the patient's well-being at the present time rather than according to future risks. Hence, giving "now" such high influence further reinforces the characteristics of today's health care, where substantial effort goes into treating patients with acute conditions. However, in general practice, most patients with acute illnesses will eventually recover, while many patients with chronic conditions experience a continuing, often gradual, decline in well-being.

In the process of priority setting, neglecting to integrate future risks and benefits in the assessment tends to underestimate the severity of the condition and the effectiveness of an intervention in patients with chronic conditions. Hence, there is a risk that the time perspective will further amplify the imbalance between chronic and acute diseases.

A question for further research is whether the three new dimensions identified at the individual level are also relevant at the national or organisational level. Are different priority-setting criteria needed at different levels? Knowledge about what weight is actually given to the key criteria in decisions concerning different health conditions and interventions could be useful in the process of developing tools for priority setting and drawing up guidelines that are perceived as useful in PHC. Further research in this area would be beneficial.

## Conclusions

Nurses and GPs perceived the three key priority-setting criteria (severity, patient benefit, and cost-effectiveness) to be valuable for priority setting in primary health care. However, three additional dimensions were identified: 1) viewpoint (medical or patient's), 2) timeframe (now or later), and 3) evidence level (group or individual). Considering these dimensions is probably a prerequisite for the criteria to be useful in priority setting for individual patients.

## Competing interests

The authors declare that they have no competing interests.

## Authors' contributions

All authors were involved in planning the study. EA and MA conducted the focus group sessions, and analysis. All authors performed the final analysis, and were involved in drafting the manuscript as well as the final approval of the manuscript

## Pre-publication history

The pre-publication history for this paper can be accessed here:

http://www.biomedcentral.com/1471-2296/11/71/prepub
